# Localization is the key to action: regulatory peculiarities of lncRNAs

**DOI:** 10.3389/fgene.2024.1478352

**Published:** 2024-12-16

**Authors:** Joice de Faria Poloni, Fábio Henrique Schuster de Oliveira, Bruno César Feltes

**Affiliations:** Department of Biophysics, Laboratory of DNA Repair and Aging, Institute of Biosciences, Federal University of Rio Grande do Sul, Porto Alegre, Rio Grande do Sul, Brazil

**Keywords:** long non-coding RNA, lncRNA, subcellular localization, tissue-specific expression, cis- and trans-acting elements, bioinformatics tools and databases

## Abstract

To understand the transcriptomic profile of an individual cell in a multicellular organism, we must comprehend its surrounding environment and the cellular space where distinct molecular stimuli responses are located. Contradicting the initial perception that RNAs were nonfunctional and that only a few could act in chromatin remodeling, over the last few decades, research has revealed that they are multifaceted, versatile regulators of most cellular processes. Among the various RNAs, long non-coding RNAs (LncRNAs) regulate multiple biological processes and can even impact cell fate. In this sense, the subcellular localization of lncRNAs is the primary determinant of their functions. It affects their behavior by limiting their potential molecular partner and which process it can affect. The fine-tuned activity of lncRNAs is also tissue-specific and modulated by their *cis* and *trans* regulation. Hence, the spatial context of lncRNAs is crucial for understanding the regulatory networks by which they influence and are influenced. Therefore, predicting a lncRNA’s correct location is not just a technical challenge but a critical step in understanding the biological meaning of its activity. Hence, examining these peculiarities is crucial to researching and discussing lncRNAs. In this review, we debate the spatial regulation of lncRNAs and their tissue-specific roles and regulatory mechanisms. We also briefly highlight how bioinformatic tools can aid research in the area.

## 1 Introduction

Eukaryotic transcriptome encompasses a broad diversity of RNA types that differ in functionality, biogenesis, compartment of activity (i.e., nucleus, cytoplasm, etc.), and tissue-specific roles and regulation mode (*cis* or *trans*). Up to 90% of the human genome was found to be transcribed during the eukaryotic life cycle; however, from this total, less than 2% correspond to protein-coding genes (Hatje et al., 2019; Scherrer, 2023). The remaining functional transcriptome comprises ncRNAs categorized as housekeeping or regulatory. Housekeeping ncRNAs are abundant in all cell types, showing constitutive expression, and are associated with regulating primary cellular function (Zhang P. et al., 2019). Among the most known housekeeping ncRNAs are ribosomal RNAs (rRNAs), transfer RNAs (tRNAs), small nuclear RNAs (snRNAs), and small nucleolar RNAs (snoRNAs) (Zhang P. et al., 2019). In turn, regulatory ncRNAs act in several biological processes, such as chromatin remodeling, transcription and translation regulation, and mRNA processing and decay (Yotsukura et al., 2017; Diamantopoulos et al., 2018; Rincón-Riveros et al., 2021) and their biogenesis and activity might be tissue- or compartment-specific. The most known regulatory ncRNAs comprise microRNA (miRNA), endogenous short interfering RNA (endo-siRNA), PIWI-interacting RNA (piRNA), long non-coding RNA (lncRNA), and circular RNA (circRNA) (Loganathan and Doss C, 2023).

LncRNAs are integral to the regulation of biological processes and play significant roles in the etiology of various pathologies, such as cardiovascular, neurodegenerative, and autoimmune diseases, neuroinflammation, and found to be deregulated in several cancer types (Lanzós et al., 2017; Diamantopoulos et al., 2018; Poller et al., 2018; Lodde et al., 2020; Ruffo et al., 2021; Chen et al., 2022; Jiang et al., 2022; Anilkumar et al., 2024).

The pervasive presence of lncRNAs underscores their indispensability in unraveling the intricacies of gene regulation. Despite the detailed characterization of specific lncRNAs, a substantial proportion still needs to be associated with biological processes and their molecular targets. According to GENCODE, the human genome has more than 20,000 lncRNA genes (Frankish et al., 2023), but just a small proportion of lncRNAs has been functionally characterized, about ∼500–1,500 (Carlevaro-Fita and Johnson, 2019). Consequently, elucidating direct regulatory targets and linking them to biological functions poses considerable complexity. This gap emphasizes the critical role of bioinformatics tools and the need for additional experimental data to predict and analyze lncRNA structure, function, and other attributes. The advancement of high-throughput techniques has markedly expedited lncRNA research and facilitated the establishment of numerous publicly accessible databases and tools to uncover lncRNA functions and interactions. Bioinformatic tools are indispensable for studying lncRNAs, providing comprehensive methods for their identification, functional prediction, structural analysis, and clinical application. These tools have revolutionized our understanding of ncRNAs, revealing their complexity and significance in gene regulation.

However, to reveal the biological role of lncRNAs, cellular localization remains a crucial factor in addressing their function. LncRNA activities are localization-specific in several ways: they are more tissue-specific than mRNA, show particular subcellular locations, and have an action mechanism that can be performed in *cis*- or *trans*-regulator. Identifying if a lncRNA is expressed within a particular tissue or if the target mature RNA is in a determined subcellular compartment is crucial for future studies involving these molecules. Furthermore, lncRNA localization has practical implications in studies that have proposed using lncRNA as biomarkers or drug targets.

This review highlights the mechanistic spatial insight of lncRNA action considering tissue expression, subcellular location, and mode of action. We also discuss the importance of bioinformatic tools in lncRNA research, demonstrating how this field has revolutionized our understanding of these non-coding elements.

## 2 Long non-coding RNA

One of the first studies to identify a lncRNA was published in 1990 by Brannan et al., where the authors discovered that the gene H19 was not translated to protein, even though this gene possesses a small open reading frame (Brannan et al., 1990; Jarroux et al., 2017). H19 has some characteristics compatible with mRNAs, such as gene transcription by RNA Polymerase II, splicing, 3′ polyadenylation, and translocation to the cytoplasm (Brannan et al., 1990). Although H19 gene expression is essential to embryonic development, only in 1990 the function of H19 was fully understood when Xist was also characterized, revealing that both lncRNAs are involved in genomic imprinting and dosage compensation in mammals (Jarroux et al., 2017).

LncRNAs have a sequence length above 200 nucleotides and a structure similar to mRNAs. However, they lack the open reading frame (ORF) or show small ORFs (length less than 300 nucleotides), and consequentially, they typically are not able to produce a full-length protein (Gong et al., 2021; Feng et al., 2023). In the case of lncRNA showing sORFs, they may synthesize small and stable micropeptides. However, a significant micropeptide fraction is unstable and degraded after synthesis (Singh, 2024). LncRNAs were considered transcriptional noise because they have low expression and show more specific tissue expression than coding protein genes (Gloss and Dinger, 2016; Feng et al., 2023).

The emergence of lncRNA research can be attributed to their regulatory functions in several biological processes. Like most genes, lncRNA-associated genes are composed of introns and exons, mainly transcribed by RNA polymerase II (Pol II) and less frequently by other polymerases (Statello et al., 2021; Mattick et al., 2023). The transcribed product being is processed by featuring 7-methyl guanosine (m7G) caps at their 5′ ends and polyadenylated tails at their 3′ ends (Khan et al., 2021; Statello et al., 2021). In addition, lncRNAs are subject to alternative or constitutive splicing after transcription. However, pre-lncRNAs are less efficiently spliced than pre-mRNA of protein-coding genes, possibly due to differences in consensus sequences for the branch point and 5′ and 3′ splice sites or interaction of specific splicing factors that can decrease alternative usage of particular exons (Khan et al., 2021). Also, lncRNA transcripts are less abundant than mRNA, which could be related to unspliced and low-stability transcripts due to an absence of proximal RNA polymerase II phosphorylation over the lncRNA 5′ splice site (Khan et al., 2021).

In contrast to what was first believed, only a minority of lncRNAs are unstable (Bridges et al., 2021). Most are stabilized through polyadenylation, and the non-polyadenylated shows secondary structures that ensure their stability (Bridges et al., 2021). In addition, it was presumed that lncRNAs are transcribed and processed the same way as mRNA; however, distinct cellular fates and functions can be related to specific coordination of lncRNA transcription, processing, exportation, and turnover (Statello et al., 2021). Some lncRNAs are transcribed by RNA polymerase I (Pol I) and RNA polymerase III (Pol III), not being polyadenylated nor receiving m7G caps, or yet resulting from the processing of different precursors, such as introns and repetitive elements (Mattick et al., 2023). In addition, many lncRNAs are successfully spliced and exported to the cytoplasm, while others are inefficiently processed during splicing, and the product is retained in the nucleus (Statello et al., 2021). In this case, a study found that due to their higher transcript complexity (i.e., multiple splice variants per exon, shorter intron length, predominant dinucleotide on splicing sites, and low conservation at 5′ and 3′ splicing sites), lncRNA splicing is more inefficient than mRNA (Basu et al., 2023). Intronic lncRNAs and unspliced lncRNAs are less stable than intergenic, cis-antisense, and spliced lncRNA (Clark et al., 2012). These lncRNA are submitted to regulators of lncRNA turnover and directed to degradation via different mechanisms, which are believed to be similar to those controlling mRNA turnover, such as decay-promoted by RBPs, microRNAs, decapping and deadenylation followed by exo- and endonucleolytic degradation and translation-associated RNA decay (Yoon et al., 2015). Likewise, despite our knowledge of which factors play a role in lncRNA decay, how this process is regulated and the impact on bioprocess is still to be elucidated (Singh, 2024). According to a study conducted in mouse Neuro-2a cell line, 29% of lncRNA were unstable (considering a half-life of 2 h or less), and 6% were highly stable (with a half-life superior to 16h) (Clark et al., 2012). Nevertheless, it should be noted that knockout of some lncRNA does not affect the observable phenotype (Gao et al., 2020), suggesting that due to their higher decay rate, they might exert no function at all, and only a selected few might truly impact cell biology (Ponting and Haerty, 2022). Despite that, a significant fraction of lncRNAs have nuclear localization signals and remain in the nucleus to exert their roles, which may influence gene expression, nuclear organization, phase separation, compartment formation, and modulating epigenetic regulation (Guh et al., 2020).

**FIGURE 1:**
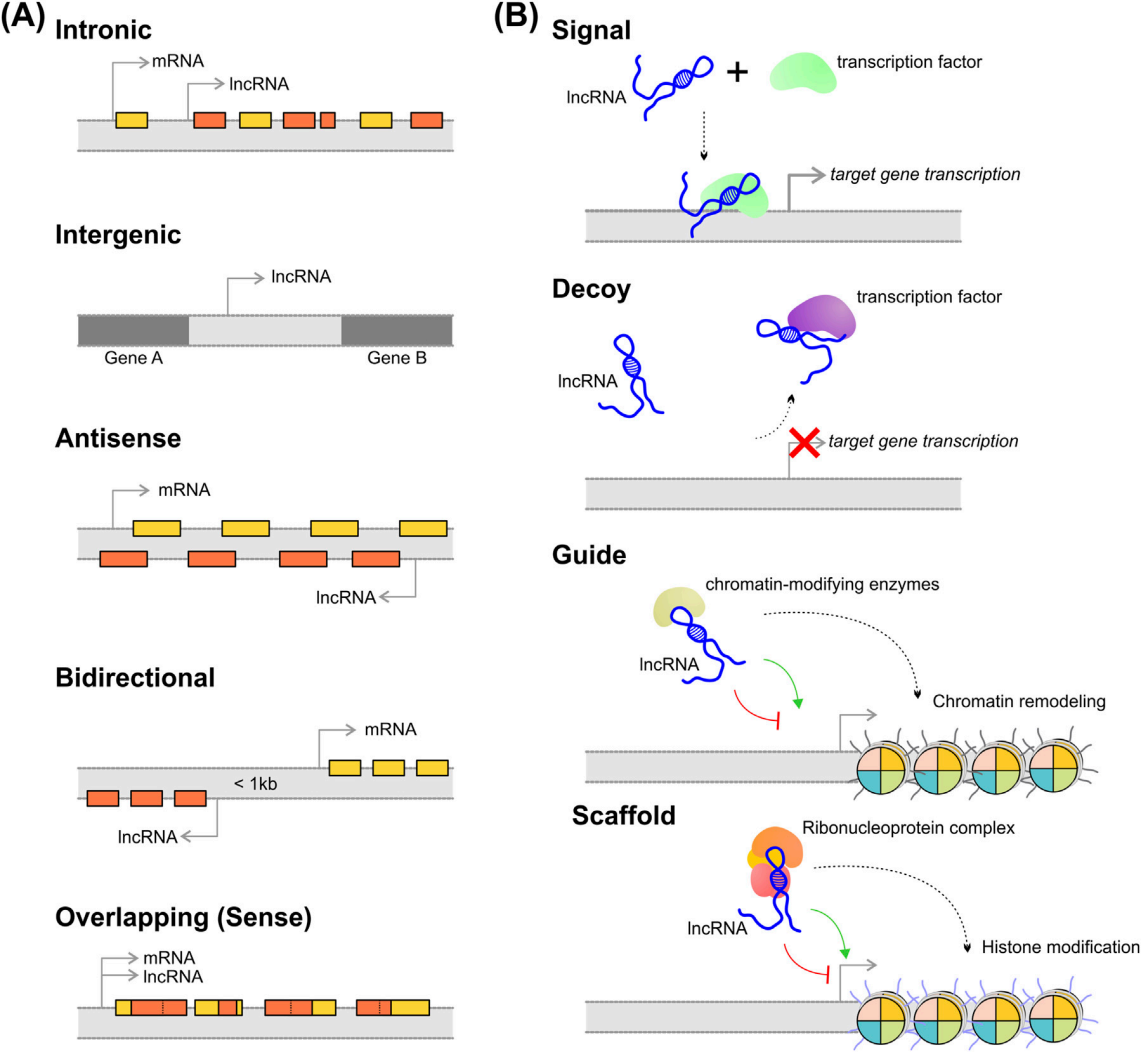
Classification and molecular function of lncRNAs. **(A)** lncRNAs classification based on their genomic position and orientation relative to nearby protein-coding genes; **(B)** lncRNAs can execute different molecular functions depending on their mechanism of action. See the main text for the explanation.

The lncRNA classification that is more frequently used was proposed by GENCODE and is based on the genomic context considering their position and orientation relative to protein-coding genes (Antonov et al., 2019). Consequently, lncRNAs may be categorized into six classes (Figure 1A): (i) intronic, referring to lncRNAs that derived from intronic sequences of protein-coding genes; (ii) intergenic, is referred to lncRNA transcribed from regions located between protein-coding genes and do not overlap with known coding sequences; (iii) antisense, the lncRNA is transcribed from the opposite strand relative to protein-coding genes; (iv) bidirectional, when the lncRNA is transcribed from promoters located in the opposite direction of neighboring protein-coding genes and often share the same promoter region; (v) overlapping (or sense), represents lncRNAs whose gene sequence overlaps another gene on the same strand.

Furthermore, lncRNAs can also be classified according to their action mode, which is closely related to their molecular function. The action mode for lncRNAs can be checked in Figure 1B and is described as (i) signal mode, which refers to lncRNA that promotes chromatin architecture modifications, silencing their target genes, or could act as an enhancer, promoting the transcriptional machinery recruitment to induce the transcription of target genes in response to different stimuli; (ii) decoy mode, refers to the lncRNA that bind to proteins with regulatory functions, such as transcription factors, and decrease the accessibility of these proteins by “sequestering” them. In this case, decoy lncRNA indirectly acts by activating or inhibiting the transcription of target genes; (iii) guide mode, where the lncRNA directs the assembly of chromatin-modifying enzymes by binding to the molecules and guiding them to specific genomic loci; (iv) scaffold mode, where the lncRNA act as a platform for the assembly and interaction of multiprotein complexes and RNA-binding factors (Ahmad et al., 2016; Gong et al., 2021).

lncRNAs have been observed to regulate bioprocesses, such as epigenetic, transcriptomic, post-transcriptional, translation, and post-translational modification (Zhang X. et al., 2019). Although evidence supporting the functionality of most lncRNAs is still missing, there is well-documented evidence that an increasing number of lncRNAs perform critical cellular roles. In part, this difficulty is related to the fact that many lncRNA are reported to be tissue-specific or even cell lineage-specific (Kashi et al., 2016; Mattick et al., 2023). For example, research found that lncRNAs have tissue-specific expression and distinct subcellular localization patterns and are expressed in narrower time windows than mRNAs along the Zebrafish developmental time course (Pauli et al., 2012).

Furthermore, different reasons make the elucidations about lncRNAs more complicated, such as the lower expression than mRNAs, making their identification challenging to detect amidst the transcriptome. Another challenge is that most studies use oligo (dT) primers for cDNA synthesis, challenging the investigation of non-polyadenylated lncRNA (Kashi et al., 2016). LncRNA also shows low overall sequence conservation across different species compared to protein-coding genes, even though their promoters, exon structure, and splice junctions infer selective constraint (Ponting et al., 2009; Kashi et al., 2016; Mattick et al., 2023). Despite the low overall sequence conservation, many lncRNAs retain preserved functions. While this is, in part, justified by the conservation of a sequence subset, it also indicates that the primary sequence of lncRNAs may not be the determinant of how they exert their function - instead, their functionality could be explained by the formation of secondary structures and specific sequence motifs that remains preserved (Ponting et al., 2009; Ulitsky and Bartel, 2013).

## 3 LncRNA landscape

### 3.1 Subcellular localization

Understanding the subcellular location of lncRNA is the primary determinant that infers its biological function since it interacts with proteins, DNA, and other RNA types. LncRNA traffic to exact subcellular locations is a process that demands energy; thus, their transport to the correct destination is relevant for the proper execution of their function (Lee et al., 2019). If this process is halted or abolished, it would impede lncRNAs from finding their interaction partners and regulating their cellular behavior correctly. Otherwise, they could engage in deleterious interactions or activities (Lee et al., 2019). Due to the importance of lncRNA spatial localization in understanding their partners and participation in different bioprocesses, several databases offering subcellular localization information were developed and explored in Table 1.

**TABLE 1:** Tools were curated manually mainly through relevant term searches on multiple academic research databases and also through cross-citation in lncRNA-related papers. Only resources updated or released since 2015 and had a working platform at the moment of curation were added to the Table. All data was collected from 17 June 2024, to 26 July 2024.

Resource	Evidence	Spatial Information	Last update	Supported species	Website link	References
Annocript	Prediction-based	-	2018	-	https://github.com/frankMusacchia/Annocript	Musacchia et al. (2015)
AnnoLnc2	Experiment and prediction-based	Tissue specific and subcellular localization	2020	Human, mouse	http://annolnc.gao-lab.org/	Ke et al. (2020)
CANTATAdb	Experiment-based	-	2024	108 plant species	http://yeti.amu.edu.pl/CANTATA/	Szcześniak and Wanowska (2024)
CPC2	Prediction-based	-	2017	-	https://cpc2.gao-lab.org/	Kang et al. (2017)
CPPred	Experiment-based	-	2019	-	http://www.rnabinding.com/CPPred	Tong and Liu (2019)
DeepLGP	Experiment-based	-	2019	-	https://github.com/zty2009/LncRNA-target-gene	Zhao et al. (2020)
DeepLncLoc	Prediction-based	Subcellular localization	2022	-	http://bioinformatics.csu.edu.cn/DeepLncLoc/	Zeng et al. (2022)
DIANA-LncBase*	Experiment and prediction-based	Tissue specific and Subcellular localization	2019	Human, mouse	www.microrna.gr/LncBase	Karagkouni et al. (2020)
ENCORI/starBase*	Experiment-based	-	2023	23 species, including human	https://rnasysu.com/encori	Li et al. (2014)
exoRBase 2.0*	Experiment-based	Tissue specific	2021	Human	http://www.exoRBase.org	Lai et al. (2022)
GENCODE*	Experiment and prediction-based	-	2023	Human, mouse	https://www.gencodegenes.org	Frankish et al. (2019)
GTEx*	Experiment-based	Tissue specific	2023	Human	https://gtexportal.org/	Lonsdale et al. (2013)
iLoc-lncRNA	Experiment and prediction-based	Subcellular localization	2018	-	http://lin-group.cn/server/iLoc-LncRNA	Su et al. (2018)
iLoc-lncRNA (2.0)	Prediction-based	Subcellular localization	2022	-	http://lin-group.cn/server/iLoc-LncRNA(2.0)/	Zhang et al. (2022)
intaRNA	Prediction-based	-	2017	-	http://rna.informatik.uni-freiburg.de/IntaRNA	Mann et al. (2017)
Lnc2Cancer 3.0*	Experiment and prediction-based	Subcellular localization	2020	Human	http://bio-bigdata.hrbmu.edu.cn/lnc2cancer	Gao et al. (2021)
LncACTdb*	Experiment and prediction-based	Subcellular localization	2022	25 species, including human	http://bio-bigdata.hrbmu.edu.cn/LncACTdb/	Wang et al. (2022)
lncADeep	Prediction-based	-	2018	-	https://cqb.pku.edu.cn/zhulab/info/1006/1160.htm	Yang et al. (2018)
LnCaNet	Experiment-based	-	2016	Human	http://lncanet.bioinfo-minzhao.org/	Liu and Zhao (2016)
lncATLAS	Experiment-based	Subcellular localization	2017	Human	https://lncatlas.crg.eu/	Mas-ponte et al. (2017)
LNCBook	Experiment and prediction-based	-	2022	Human	https://ngdc.cncb.ac.cn/lncbook	Li et al. (2023)
lnCeRBase*	Experiment-based	-	2018	Human	http://www.insect-genome.com/LncCeRBase	Pian et al. (2018)
LncExpDB	Experiment and prediction-based	Tissue specific and subcellular localization	2021	Human	https://bigd.big.ac.cn/lncexpdb	Li Z. et al. (2021)
lncFunTK	Experiment-based	-	2018	-	https://github.com/zhoujj2013/lncfuntk	Zhou et al. (2018)
LNCipedia	Experiment-based	-	2018	Human	https://lncipedia.org	Volders et al. (2019)
lncLocation	Prediction-based	Subcellular localization	2020	-	https://github.com/FengSY-JLU/Core-lncLocation/	Feng et al. (2020)
lncLocator 2.0	Prediction-based	Subcellular localization	2021	-	www.csbio.sjtu.edu.cn/bioinf/lncLocator2	Lin et al. (2021)
LncRNA2Target*	Experiment-based	-	2021	Human, mouse	http://bio-computing.hrbmu.edu.cn/lncrna2target/	Cheng et al. (2019)
lncRNAdb	Experiment-based	Subcellular localization	2015	10 species, including human	https://rnacentral.org/expert-database/lncrnadb	Amaral et al. (2011)
lncRNADisease v3.0*	Experiment and prediction-based	-	2023	4 species, including human	http://www.rnanut.net/lncrnadisease	Lin et al. (2024)
lncRNASNP2*	Experiment and prediction-based	-	2018	Human, mouse	https://guolab.wchscu.cn/lncRNASNP//#!/	Miao et al. (2018)
lncRNAWiki 2.0	Experiment-based	Subcellular localization	2021	Human	https://ngdc.cncb.ac.cn/lncrnawiki	Liu et al. (2022)
LncRRIsearch	Experiment and prediction-based	Tissue specific and subcellular localization	2019	Human, mouse	http://rtools.cbrc.jp/LncRRIsearch/	Fukunaga et al. (2019)
LncSEA	Experiment-based	-	2021	Human	http://bio.liclab.net/LncSEA/index.php	Chen et al. (2021)
LncSpA	Experiment-based	Tissue specific	2019	Human	http://bio-bigdata.hrbmu.edu.cn/LncSpA	Lv et al. (2020)
LncTarD 2.0	Experiment and prediction-based	-	2022	Human	http://bio-bigdata.hrbmu.edu.cn/LncTarD	Zhao et al. (2023)
Locate-R	Prediction-based	Subcellular localization	2020	-	http://locate-r.azurewebsites.net/	Ahmad et al. (2020)
ncFANs v2.0*	Experiment-based	Tissue specific	2021	Human, mouse	http://ncfans.gene.ac/	Zhang et al. (2021)
NONCODEV5*	Experiment and prediction-based	-	2020	17 species, including human	http://www.noncode.org/	Fang et al. (2018)
NPInter v5.0*	Experiment and prediction-based	-	2022	60 species, including human	http://bigdata.ibp.ac.cn/npinter5/	Zheng et al. (2023)
RefLnc	Experiment-based	-	2017	Human	https://reflnc.gao-lab.org/	Jiang et al. (2019)
RIblast*	Prediction-based	-	2020	-	https://github.com/fukunagatsu/RIblast	Fukunaga and Hamada (2017)
RISE*	Experiment-based	-	2017	3 species, including human	http://rise.life.tsinghua.edu.cn.	Gong et al. (2018)
RNADisease v4.0*	Experiment and prediction-based	-	2022	117 species, including human	http://www.rnadisease.org/	Chen et al. (2023)
RNAenrich*	Experiment-based	-	2023	Human, mouse	http://idrblab.cn/rnaenrich/	Zhang et al. (2023)
RNAInter v4.0*	Experiment and prediction-based	-	2021	156 species, including human	http://www.rnainter.org	Kang et al. (2022)
RNALocate v3.0*	Experiment and prediction-based	Tissue specific and subcellular localization	2024	242 species, including human	http://www.rnalocate.org/	Wu et al. (2022)
SEEKR	Prediction-based	-	2024	-	https://github.com/CalabreseLab/seekr	Kirk et al. (2018)
SFPEL-lpi	Prediction-based	-	2017	-	http://www.bioinfotech.cn/SFPEL-LPI/	Zhang et al. (2018)
TANRIC	Experiment-based	-	2022	Human	https://www.tanric.org	Li et al. (2015)
TransCistor	Prediction-based	-	2024	Human and mouse	https://github.com/gold-lab/TransCistor https://transcistor.unibe.ch/	Dhaka et al. (2024)

Table legends: *supports other types of RNA besides lncRNA.

The subcellular location of RNAs is not stochastic but a dynamic and regulated process that controls the localization of protein expression, turnover, and subsequent signal regulation in response to homeostatic, stimulated, or cellular stress conditions (Bridges et al., 2021). The first observation of the asymmetrical distribution of mRNA was made in 1983 (Jeffery et al., 1983). Subsequent research and methodological advances have shed light on this non-random allocation, proposing that the distribution of RNA in a cell is directly related to local protein concentration, as a non-uniform gradient distribution of RNA may perform a regulatory function (Carlevaro-Fita and Johnson, 2019).

Several mechanisms regulate the localization of the lncRNA, which are primarily present in the nucleus and cytoplasm; however, many studies have found lncRNA present in organelles and macromolecular structures, such as mitochondria, endoplasmic reticulum, ribosomes, extracellular membrane, exosome, nucleolus, chromatin speckles, and paraspeckles (Dragomir et al., 2018; Carlevaro-Fita and Johnson, 2019; Bridges et al., 2021; Li C. et al., 2021). The enrichment of lncRNAs in specific locations is orchestrated by different sequence motifs or domains, lncRNA secondary structure, and post-transcriptional modifications (Carlevaro-Fita and Johnson, 2019). Nuclear retention may be defined by the primary sequence of lncRNA and may include short motifs, such as hexamers, structural elements, transposable element fragments, or longer sequence domains (Carlevaro-Fita and Johnson, 2019).

Most lncRNA’s Post-transcriptional modifications are similar to mRNA’s that are crucial for trafficking and nuclear exportation; hence, they share a common exporting pathway (Carlevaro-Fita and Johnson, 2019; Guo et al., 2020). In this sense, the transcription-export complex (TREX), nuclear RNA export factor 1 (NXF1), and nuclear transport factor 2-like export factor 1 (NXT1) promote the lncRNA transportation, especially by NXF1, which is preferentially used for the exportation of RNA composed by single or few exon or RNA enriched with high A/U content (Guo et al., 2020). However, lncRNA that have divergent transcription, caused by different phosphorylation pattern Pol II C-terminal domains (CTDs), or do not share the same processing of mRNA transcripts, may be inefficiently exported by the protein complexes mentioned above (Guo et al., 2020; Khan et al., 2021). This may occur by the fact that RNA processing, co-transcriptional splicing, 3′ cleavage, and polyadenylation are strongly related to CTD Ser5 and Ser2 phosphorylation; however, intergenic lncRNA showed to be less selective to CTD profile and Pol II pausing at the transcription start and end sites are generally absent on these lncRNAs (Schlackow et al., 2017).

Furthermore, chromatin marks and Pol II promoter-proximal pausing are also related to nuclear exportation, possibly allowing the association of lncRNA with exporting proteins (Zuckerman and Ulitsky, 2019). Altogether, splicing efficiency is substantially increased in lncRNA transcript found in the cytoplasm compared to those retained in the nucleus (Zuckerman and Ulitsky, 2019).

Alternatively, unusual stabilization may occur by forming structures at their ends that confer stability and ensure RNA stability and the exportation of the RNA from the nucleus (Wilusz et al., 2012). Different structures at the end of lncRNA are formed by distinct motifs and described to confer stability and direct the lncRNA to proper subcellular localization. The nascent transcript is processed at the 3′ end during transcription, ensuring the mature RNA’s functionality. Most RNA Pol II transcripts receive a poly(A) tail conceived from cleavage by endonucleases and the addition of adenosine (A) residues. This poly(A) tail confers stability and allows the mature RNA exportation from the nucleus. However, some Pol II transcripts, instead of having poly(A) tail, show a different 3′ RNA conformation, such as the triple-helical structure found in the lncRNA MALAT1, formed by highly conserved A- and U-rich motifs at 3′ end and promote nuclear retention of MALAT1 and its enrichment in nuclear speckles (Wilusz et al., 2012). Another example is SLERT, a lncRNA specially located in the nucleolus, and its localization and biogenesis are mediated by a box H/ACA at both ends (Zhang Y. et al., 2017). Different lncRNA sequence motifs may interact with several partners, such as hnRNPs, RNA helicases, and RNA processing factors, to restrict the lncRNA to the nucleus (Guo et al., 2020).

In general, cytoplasmic lncRNAs are more stable than their nuclear counterparts. In the nucleus, lncRNA regulates transcription by interacting with and remodeling chromatin and establishing the spatial organization of nuclear compartments through their scaffold function (Bridges et al., 2021). Additionally, lncRNA may interact with splicing factors and regulate splicing events (Ouyang et al., 2022). In the cytoplasm, lncRNAs play a crucial role in the post-transcriptional regulation of gene expression and influence several pathways by, for instance, acting as sponges for miRNAs, thereby regulating the expression of miRNA target genes and interacting with RBPs to modulate their activity and influence mRNA stability and translation (Noh et al., 2018; Aillaud and Schulte, 2020). However, many lncRNAs exhibit dynamic subcellular localization in response to pathological stimuli. In these contexts, the altered localization of lncRNAs can profoundly affect cellular function, gene expression, and disease outcomes. In the literature, we can find plenty of lncRNA that change location in certain diseases, but cancer is the most common. Under normal conditions, MALAT1 is primarily located in the nucleus, retained within nuclear speckles (Miyagawa et al., 2012). However, the translocation of MALAT1 from the nucleus to the cytoplasm can occur under various cellular conditions, particularly in response to cellular stress, such as oxidative stress, or during certain pathological conditions like cancer (He et al., 2018). MALAT1 has been shown to interact with microRNAs (miRNAs) in the cytoplasm, which suggests that it may function as a molecular sponge for these miRNAs. This interaction can influence the availability and activity of the miRNAs, thereby affecting gene expression and cellular processes (He et al., 2018). Nevertheless, the exact trigger for this translocation is not clear.

Another case is the lncRNA Taurine Upregulated Gene 1 (TUG1), an oncogenic lncRNA whose splicing process determines TUG1 localization. Intron retention promotes nuclear compartmentalization, while fully spliced transcripts are found in the cytoplasm (Dumbović et al., 2021). Although intron retention in nuclear TUG1 was increased in HeLa and U-2 OS cells, the functional consequences remain elusive.

Comprehending the subcellular localization of lncRNAs is not just a scientific pursuit but a potential pathbreaker in molecular biology, genetics, and bioinformatics. This knowledge is crucial for unraveling the diverse functions of lncRNAs in cellular physiology and pathology. However, this path has only begun to be traveled, and many challenges still need to be overcome due to the dynamic nature of the lncRNA and the complexity of their interactions associated with low gene expression. Also, it cannot be ignored that some of these molecules may have localization context-dependent, varying with cellular conditions, developmental stages, or disease states.

### 3.2 Lineage and tissue-specific expression

The regulatory action of lncRNA is directly related to their spatial expression across different tissues, being more tissue-specific than protein-coding gene expression (Lv et al., 2020). In this sense, many lncRNA expressions are confined or more expressed in a specific tissue while, at the same time, showing lower levels in other tissues. In this scenario, a comprehensive picture of the lncRNA landscape is essential once a dysfunction of its activity could be related to a tissue-specific pathology. While lncRNA expression levels are typically lower than mRNA, they show stronger tissue-specific spatial expression patterns, suggesting that they may have a differential role in specific cell types (Lv et al., 2020; Bridges et al., 2021). As mentioned above, lncRNA genes show less overall sequence conservation than protein-coding genes; however, their promoter sequence and transcription factor binding sites are conserved, indicating that a conserved regulatory mechanism governs lncRNA transcription (Mattioli et al., 2019).

Despite this tissue-specific expression preference, some lncRNAs are ubiquitously expressed in almost all tissues, indicating a universal housekeeping function (Jiang et al., 2016; Xu et al., 2023 showed that the testis, brain, and kidney expressed a significant fraction of lncRNAs, but only a small number were among the highly expressed genes (Xu et al., 2023). In contrast, the liver and muscle expressed fewer lncRNAs (Xu et al., 2023). More than 95% of lncRNA expressed in the muscle were among the top thousand highly expressed genes; in the testis, however, less than 63% were among the top expressed genes (Xu et al., 2023). The authors suggested that the difference observed between these tissues could be explained by the more specialized nature, and the greater the cell types number, the greater the lncRNAs proportion present in abundance (Xu et al., 2023). In addition, a comparative analysis across 94 samples and 20 tissue types showed that 1,184 lncRNAs expressed in all evaluated tissues have a higher expression level pattern than lncRNA exclusively expressed in only one tissue, suggesting that ubiquitous lncRNA may have higher expression levels than tissue-specific lncRNA (Jiang et al., 2016). Furthermore, lncRNA ubiquitously expressed tends to have the highest sequence conservation levels, fewer exons, and fewer isoform transcripts than tissue-specific expressed lncRNA (Jiang et al., 2016). This result is consistent with the observed for housekeeping protein-coding genes. Combined with these ubiquitous RNA’s higher expression levels, it suggests a selective pressure for energy conservation, resulting in a more compacted gene structure that minimizes transcription and processing energetic costs (Jiang et al., 2016). An alternative hypothesis suggests that tissue-specific protein-coding genes have a longer gene architecture due to regulatory and functional complexity because these genes show more functional domains than housekeeping genes (Vinogradov, 2004). Also, tissue-specific protein-coding genes could be subjected to optimized chromatin suppression and more complex regulation of its expression (Vinogradov, 2004). This hypothesis is still a matter of discussion, especially considering lncRNAs, where the differences between housekeeping and tissue-specific lncRNA are still under exploration.

Jiang et al. found that 76.5% of tissue-specific lncRNA genes are located in intergenic regions and are targeted by fewer transcription factors and regulatory miRNA than ubiquitously expressed lncRNA (Jiang et al., 2016). This result suggests that lncRNA ubiquitously expressed are under the strictest regulation than tissue-specific lncRNA (Jiang et al., 2016).

Mattioli et al. showed that the core promoter sequence is a determinant that explains tissue specificity. They proposed that highly abundant genes have complex and promiscuous transcription factor binding sites (Mattioli et al., 2019). The authors showed that the overlapping transcription factor binding sites are more frequent in ubiquitously expressed genes, contrasting tissue-specific genes showing fewer overlapping motifs (Mattioli et al., 2019). In addition, it was found that the intergenic lncRNA has less complex transcription factor motifs at the core promoter and fewer overlapping motifs, corroborating with the overall lower expression and higher tissue specificity described for most lncRNAs (Mattioli et al., 2019).

Another candidate that could contribute to this tissue specificity regulation observed in lncRNA is the transposable elements (TE) that could influence the lncRNA regulatory network (Kelley and Rinn, 2012; Chishima et al., 2018). One example is the long terminal repeats (LTR) of endogenous retroviruses (ERV) that could act as alternative promoters (Kelley and Rinn, 2012). LTR/ERV elements generally are silenced in most human tissues except for a subset of family members that become active in some tissue or cell types (Seifarth et al., 2005; Kelley and Rinn, 2012; Tokuyama et al., 2018). It was observed that lncRNA containing ERV1 element was more likely expressed in the testis than lncRNA containing Alu element, which was less likely expressed in this tissue (Chishima et al., 2018). Interestingly, the L1PA2 element was found to act as lncRNA promoter, driving the tissue-specific lncRNA expression specifically in the placenta (Chishima et al., 2018).

Furthermore, it is known that TE are prevalent in lcnRNAs exons relative to protein-coding gene exons, which are directly related to their composition and diversification (Chishima et al., 2018). Johnson and Guigó proposed that TE plays a fundamental role in composing functional domains in lncRNA and drives regulatory lncRNA evolution (Johnson and Guigó, 2014). In fact, several pieces of evidence have proposed TE as a functional element of the lncRNA (Kapusta et al., 2013; Chishima et al., 2018; Fort et al., 2021). A comparative analysis between primate species considering overall genes showed that genome regions associated with transcription and development are devoided of TE, possibly due to a stronger selective pressure that avoids TE inserted in genes with essential functions and their vicinity (Mortada et al., 2010). Corroborating with this matter, TE-free genes and their surrounding regions are highly conserved across primates compared to TE-rich genes. However, TE-derived lncRNA exons showed higher purifying selection than non-TE-derived sequences, suggesting that at least a subset of lncRNA sequences are under purifying selection constraints (Kapusta et al., 2013).

LncRNA exhibits spatiotemporal pattern expression and has emerged as an essential tissue physiology regulator. A study considering 1340 post-mortem human brain samples of different brain regions during distinct developmental stages showed that temporal changes in lncRNA expression across fetal development are higher than those observed in the postnatal period through late adulthood (Zhang X. Q. et al., 2017). However, fewer differences in lncRNA expression were observed during the prenatal period across different brain regions, contrasting with those found in postnatal and adult stages (Zhang X. Q. et al., 2017). In these last stages, the cerebellar cortex revealed the most distinguishable lncRNA expression compared to the mediodorsal nucleus of the thalamus, striatum, amygdala, hippocampus, and neocortex regions (Zhang X. Q. et al., 2017). Considering samples of the neurodegenerative Alzheimer’s and Parkinson’s disease, the authors showed that many lncRNA involved in these disorders were related to fetal brain development (Zhang X. Q. et al., 2017). These results prove that lncRNAs are regulated spatiotemporally, deeply influencing neurodevelopment (Zhang X. Q. et al., 2017). In fact, several lncRNA genes are upregulated in Alzheimer’s disease, such as BACE1-AS, 51A, and LRP1-AS, and the lncRNA 51A has been proposed as a biomarker to evaluate cognitive decline (Srinivas et al., 2023; Fang et al., 2024).

Exploring the landscape of ubiquitously expressed and tissue-specific lncRNA is essential to better understanding these molecules in different pathologies. More importantly, it highlights their potential as diagnostic biomarkers and therapeutic targets in various diseases.

### 3.3 *Cis* and *trans*: mechanism of action

To execute their regulatory role, lncRNAs interact with specific DNA target sequences, other RNAs, or RNA-binding proteins (RBPs), which could work in cis or trans-acting mode (Tao et al., 2023). In the cis-acting lncRNA mode, they can execute their function at the site of transcription, affecting neighboring genes, while the lncRNA that function in the trans-acting mode can affect their target at distant locations of its own transcription (Tao et al., 2023). The *cis*- and *trans*-regulatory roles of lncRNAs are fundamental to understanding the magnitude with which these molecules affect cellular biological processes and how they can interfere with pathological conditions.

*Cis*-acting lncRNA executes its regulatory function by targeting genes at the same loci as the lncRNA gene. *Cis*-acting lncRNA can act as repressors and activators in targets as distant as one hundred base pairs to hundreds of kilobases (Dhaka et al., 2024). This may occur when *cis*-regulatory lncRNAs modulate gene expression by the recruitment and/or influencing the function of regulatory factors, such as preinitiation complex formation and transcription factors (Ma et al., 2013; Zhao et al., 2024). Yet, *cis*-acting lncRNA may affect the expression of neighbor genes by different mechanisms. One way to perform their activity directly relates to regulating chromatin states by using chromatin looping or local domains created by preferential chromatin interactions called “topologically associating domains” (Dhaka et al., 2024). Also, it was proposed that *cis*-acting lncRNA promotes chromatin remodeling by recruiting chromatin modification complexes, such as the polycomb repressive complex (PRC) (Ma et al., 2013). Another mechanism involves *cis*-acting lncRNA transcribed from enhancer regions (Gil and Ulitsky, 2020). Enhancer lncRNA (e-lncRNA) have been found overlapped to enhancer regions, and it is proposed that these transcripts contribute to modulating the enhancer activity, which has been shown that enhancer-producing lncRNA has a stronger activity compared to enhancers that do not (Gil and Ulitsky, 2020). It is essential to distinguish e-lncRNA from enhancer RNA (e-RNA), which can be accomplished by their size and stability once e-RNA are shorter and unstable (Gil and Ulitsky, 2020).

In contrast, *trans*-acting lncRNAs demonstrate a versatile regulatory function, with no apparent preference for regulating targets in specific distances or positions. Similar to mRNAs, after transcription and processing, *trans*-acting lncRNAs exert their function in a destination independent of their transcription site (Gil and Ulitsky, 2020). They regulate chromatin state and gene expression, interact and regulate different biomolecules in different cellular locations, and influence nuclear structure and organization (Zhao et al., 2024). The localization of *trans*-acting lncRNAs in the cytoplasm, organelles, other cellular structures, and/or nucleoplasm supports their identification as *trans*-regulatory lncRNAs. (Zibitt et al., 2021).

The origin of new lncRNA could be the result of different mechanisms responsible for the origin and diversity of lncRNA loci, such as sequence duplication, loss of coding potential of protein-coding genes, formation of the new transcriptional unit due to the integration of TE, mutations that alter the splicing process, and mutation that contribute to the creation of a favorable combination of sequences, such as promoters, polyadenylation elements, and splice sites (Ulitsky, 2016). All these mechanisms represent possibilities for the emergence of a new lncRNA from a locus previously transcriptionally silent (Gil and Ulitsky, 2020). In addition, it has been suggested that enhancer and promoter regions could be favorable sources for novel transcripts (Gil and Ulitsky, 2020; Palazzo and Koonin, 2020). It is hypothesized that lncRNA derived from protein-coding genes are more likely to act in *trans* than enhancer-derived lncRNA, which is more likely to act in *cis* (Gil and Ulitsky, 2020). Interestingly, 30%–60% of transcribed lncRNA are derived from regions with enhancer characteristics, and their transcription start sites tend to overlap these regions (Gil and Ulitsky, 2020). However, besides these lncRNAs initially showing a *cis*-activity by promoting chromatin remodeling and locally coordinating gene expression, it is proposed that progressively over time, *cis*-acting lncRNA become *trans*-regulators or, in some cases, carry both functions (Gil and Ulitsky, 2020; Palazzo and Koonin, 2020).

An important subset of lncRNAs is the non-coding natural antisense transcripts (NATs) that can be defined as transcripts originating from the antisense DNA strand of coding or non-coding sense transcripts (Balbin et al., 2015; Werner et al., 2024). These ncNATs are known to act as regulatory components of gene expression by either cis-acting, which refers to ncNATs that regulate their sense counterparts, or trans-acting, which originate from genomic regions that differ from their regulatory targets’ origins. The dysregulation of ncNATs can be observed in multiple human diseases such as breast cancer (Iranpour et al., 2016), Angelman’s disease (Meng et al., 2012), and Alzheimer’s (Faghihi et al., 2008), indicating that these regulatory molecules could play an essential role in distinct diseases. On the subject of spatial information concerning ncNATs, it is essential to note that, as observed in other types of lncRNA, ncNATs' transcription levels can fluctuate significantly across different tissues and cell types (Werner et al., 2007; Balbin et al., 2015). Antisense RNAs can be found both in the nucleus and cytoplasm but tend to be concentrated in the nucleus (Lee et al., 2014). Finally, general information on ncNATs is still limited, given that the field of study on these molecules is still highly recent. Yet, the current advances in this area hint at optimistic perspectives on developing ncNATS comprehensive knowledge and its applications in science. We recommend the following reviews for more detailed information on ncNATS (Wight and Werner, 2013; Krappinger et al., 2021; Werner et al., 2024), as it is not the main focus of this review.

## 4 Investigating the lncRNA world with bioinformatics tools

The post-genomic era is marked by the constant development of techniques and increased analytic complexity. This term described the myriads of omics science that emerged after human genome sequencing. Altogether, the advancement of large-scale technologies has enabled the growing generation of data that allows advances in molecular characterization under different conditions and pathologies. This colossal generation of data is attractive to the advancement of research and valuable material for studying different molecules, which could have been initially ignored by the original research, such as lncRNAs. In this sense, bioinformatics has been representing an area of paramount importance, playing an essential interdisciplinary role in the development of methodological approaches to decipher and understand the massive amount of data generated, integrating and organizing different biological evidence. However, although many lncRNAs were well-characterized, the function of most lncRNAs remains unknown or poorly understood. In addition, the interest in lncRNA emerged only in the last decade and has been a source of debate. Different approaches have emerged with the fast advance of next-generation sequencing to solve this question. LncRNA-centric methods are used to investigate the interactions between known lncRNA and their molecular partners, such as RNA pull-down and ChIRP (chromatin isolation by RNA purification) (Tao et al., 2023). In this situation, preliminary experimentation is necessary to determine a specific lncRNA of interest and, consequently, if there is a limited detection capacity. For this reason, more methods focused on throughput approaches have been explored to comprehend better lcnRNA and their interactions with RNA, DNA, and proteins (Tao et al., 2023).

**FIGURE 2:**
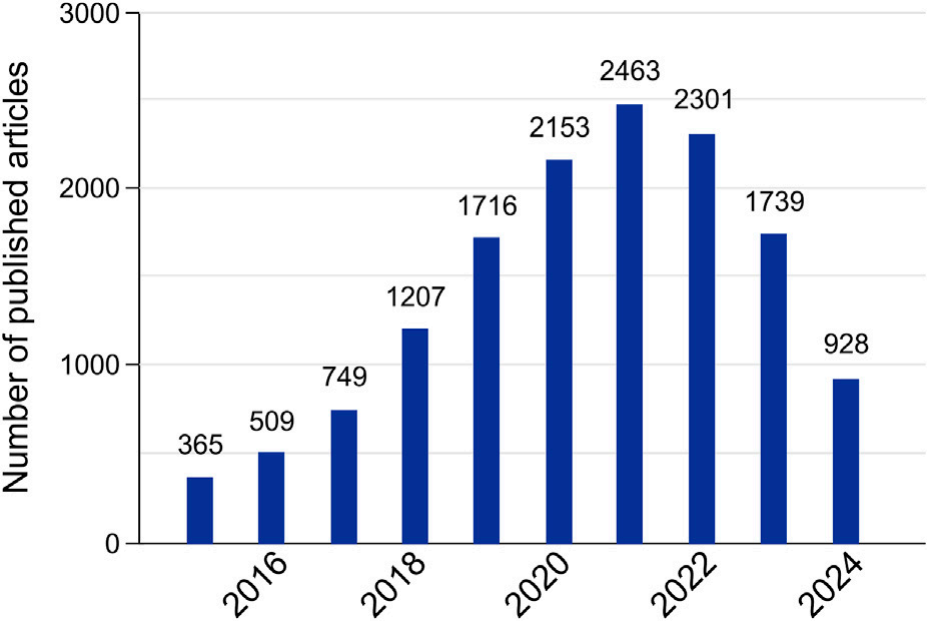
Number of lncRNA tools published by year. The chart was made by searching for lncRNAs-associated databases and computational resources using a combination of the keywords “lncRNA tools,” “lncRNA bioinformatics,” “lncRNA software,” “lncRNA database,” and “lncRNA algorithm” in the PubMed database. Although the chart only shows the resources from 2015 onward, our search was performed from 17 June 2024, to July 26th of 2024. We only included 2015 onwards to match the years where up-to-date tools (from Table 1) were created.

In the last few years, there has been a great increase in the research interest regarding lncRNA, as observed in the graph in Figure 2. With that, numerous bioinformatics resources dedicated to studying lncRNA have been developed. In this work, we compiled some of these tools that have been updated/released since 2015. The resources are collected in Table 1.

In general, most of the investigated tools were developed after 2015, and only a small portion of the ones released in earlier years have not been updated after 2015. This corroborates with the crescent research interest in lncRNA and the consequent increased demand for computational resources that may assist such studies. Additionally, it is worth mentioning that a significant number of the tools, especially databases, have received regular updates since their launch.

We also found that approximately 40% of the resources are not specific to lncRNAs and support other types of ncRNA. A considerate portion of these are tools developed for the general area of ncRNAs and, therefore, contain some information on or support for lncRNA.

When it comes to the species contemplated in each of these computational resources, we observed that most of them support few species, and, in most cases, these few species include humans. This sort of “bias” most likely results from the known role of lncRNAs in the pathogenesis of numerous human diseases and their medical importance as biomarkers and therapeutic targets. Furthermore, it is also notable that a few resources support plants despite the recently unveiled importance of lncRNA in various regulatory processes in the organisms of this taxa.

AnnoLnc2, LncExpDB, and RNAlocate (Table 1) represent metasearch resources with multi-functionalities that include tissue-specific expression and subcellular location (Ke et al., 2020; Li Z. et al., 2021; Wu et al., 2024). In addition, AnnoLnc2 extends the information and offers data regarding regulation and partner interaction, secondary structure and genomic location, genetic association, and sequence conservation (Ke et al., 2020). Otherwise, despite Lnc2Cancer3.0 and DIANA-LncBaseV3 (Table 1) being repositories focused on human cancer and miRNA targets, respectively, both databases show relevant and interesting data about lncRNA that could complement or support information about the expression profile of lncRNA in different cell types and tissues (Karagkouni et al., 2020; Gao et al., 2021). In addition, DIANA-Lnc2Cancer3.0 offers user survival analysis, correlation analysis, and transcription factor motif prediction that could be very helpful depending on the user project context (Gao et al., 2021).

When we consider prediction tools or databases to unravel the lncRNA subcellular localization, we found numerous resources that could help investigate this matter (Table 1). Several experimental methods can be used to map lncRNA to their cell compartments, such as fluorescence *in situ* hybridization (FISH), fluorescent *in situ* sequencing (FISSEQ), and subcellular RNA sequencing (subcRNAseq), which only the least is capable of potentially mapping whole transcriptome (Mas-ponte et al., 2017). LncATLAS (Table 1) is a comprehensive database that provides information on lncRNA localization that is expressed in units of Relative Concentration Index (RCI), which represents the comparison between two cellular compartments (Mas-ponte et al., 2017). It provides valuable information based on experimental data prevenient from RNA-seq datasets from 15 cell lines containing information about subcellular localization. In this sense, these databases are limited by the lncRNA captured in previous experiments. In contrast, prediction tools such as LncLocator, DeepLncLoc, and iLoc-lncRNA (Table 1) use the primary sequence of lncRNA to infer the subcellular localization using a benchmark dataset (Su et al., 2018; Lin et al., 2021; Zeng et al., 2022). These tools use machine learning approaches, such as SVM + Randon Forest, SVM, and Deep Neural Network, respectively, inputted by k-mers features to achieve a decent performance in predicting subcellular localization of lncRNAs (Zeng et al., 2022).

Predictive analysis of lncRNA localization involves complex algorithms that estimate the compartment distribution based on sequence features, interactions, and expression patterns. However, these models often face limitations in their ability to accurately predict localization, particularly for novel or poorly characterized lncRNAs. Additionally, the development and optimization of computational tools must balance computational efficiency and the ability to provide detailed and accurate localization predictions. The quality of input data can influence the accuracy of predictions, the quality of benchmarks used during model training, and the sophistication of the underlying algorithms.

Investigating whether a lncRNA acts in a *cis*- or *trans*-mode involves utilizing various bioinformatic tools and approaches to elucidate its functional mechanisms. To determine the mode of action of lncRNAs, researchers employ a combination of computational tools and databases to analyze expression patterns, chromatin interactions, and genetic perturbations. However, in most cases, several studies use methods to infer *cis*- or *trans*-regulation based on indirect relations. For instance, bioinformatic tools like LncRNA2Target (Table 1) facilitate the prediction of lncRNA-target interactions (Cheng et al., 2019). LncRNA2Target is a curated database to investigate the lncRNA-target partnership using mainly lncRNA knockdown and overexpression experiments, luciferase reporter assays, immunoprecipitation assays, and RNA pull-down assays as a source of information (Cheng et al., 2019). Dhaka et al. developed the framework TransCistor (Table 1) that proposed elucidating *cis*-regulatory relationship by using loss-of-function perturbations from FANTOM6 repositories to identify target genes of lncRNA (Dhaka et al., 2024). The author considers a molecule as a lncRNA target when the steady-state level of this molecule is significantly altered in response to the loss-of-function of the lncRNA (Dhaka et al., 2024). Another approach used is to evaluate the gene co-expression analysis to compare the gene expression between the lncRNA and their neighbor to infer if the target expression could be influenced by lncRNA (Li et al., 2017; Wu et al., 2019). Thus, this approach can help identify potential *cis*-acting targets by examining whether lncRNAs and their target genes are co-expressed and physically proximal in the genome.

Additionally, Chromosome Conformation Capture coupled with High Throughput Sequencing (Hi-C), Hi-C coupled with Chromatin Isolation by RNA Purification (Hi-ChIRP), and Chromatin Interaction Analysis by Paired-End Tag Sequencing (ChIA-PET) are examples of methods that provide insights into the three-dimensional interactions between lncRNAs and genomic regions, revealing potential *cis*- or *trans*-acting relationships (Saxena and Carninci, 2011; Ariel et al., 2021). These approaches help determine whether lncRNAs interact with nearby or distant genomic regions.

Despite these advancements, predicting lncRNA localization remains complex due to the dynamic nature of lncRNA interactions and cellular environments. Future developments are expected to focus on integrating multi-omic data and improving model accuracy. The complexity of the transcriptome, including the presence of multiple isoforms across tissues and overlapping regions between lncRNAs and protein-coding genes, increases the difficulty of finding a solution.

Furthermore, public databases and repositories may have incomplete or inconsistent data coverage across different tissues. Available datasets often represent only a limited subset of tissues, which may only partially capture the tissue-specific expression profiles of lncRNAs. This limitation can hinder identifying and characterizing lncRNAs with unique or restricted expression patterns. Variability in data quality due to differences in sample preparation, sequencing platforms, and experimental protocols can impact the reliability of expression measurements and subsequent analyses.

Investigating lncRNA localization or tissue-specific expression typically involves integrating various types of omics data, such as transcriptomic, proteomic, and imaging data. The differences in data formats, resolutions, and scales complicate the integration of all this information. Bioinformatics tools must accurately differentiate between isoforms accounting for their specific localizations and effectively combine these disparate data types to provide a coherent scenario of lncRNA. The challenges lie in developing algorithms that integrate multi-dimensional data while minimizing artifacts and maintaining biological relevance.

## 5 Conclusion

Given their low overall expression and tissue/cell-line specificity, the understanding of lncRNAs remains complex. Localizing lncRNAs within the subcellular compartments is a crucial determinant of their molecular functions, playing a significant role in cellular processes. As lncRNA can interact with a wide range of molecules, their localization and local partner interaction are crucial to understanding and predicting their function (Bridges et al., 2021). Based on the experimental information in public repositories associated with *in silico* prediction techniques, computational tools can be a differential approach to guide experiments to evaluate a hypothesis, saving time and financial resources. However, ideally, different experimental validation approaches are necessary to prove the findings, such as fluorescent in-situ hybridization (FISH)-based methods and MS2-system-based techniques (Choudhury et al., 2024). Ultimately, further studies focused on specific cases will be needed to expand our current, albeit incomplete, knowledge of lncRNA biology. Fortunately, new-generation technologies and bioinformatics tools have advanced our understanding of these regulatory molecules. To further expand our knowledge, future research should focus on elucidating the specific mechanisms by which lncRNAs regulate gene expression and cellular processes context-dependently. This will involve investigating the intricate interplay between lncRNAs and other cellular components, such as proteins, DNA, and other RNA species, and how these complex interactions contribute to lncRNA-mediated regulation of cellular homeostasis and disease pathogenesis.
